# Supplementary material for: Comparison of Adenine-Induced Rat Models for Vascular Calcification in Chronic Kidney Disease
Source: Biology (Basel). 2025 Jul 4;14(7):814. doi: 10.3390/biology14070814 (PMC12292064; doi:10.3390/biology14070814)
Supplement: Supplementary file 1 [file biology-14-00814-s001.zip › biology-3669907-supplementary.pdf]

Supplementary Table S1. Body weight and serum biochemistry at 18 wk.

Parameters	Control (n = 12)	Group 1 (n = 12)	Group 2 (n = 12)	Group 3A (n = 12)	Group 3B (n = 12)	Group 3C (n = 12)
**Body weight (g)**	513.8 ± 37.1	545.6 ± 12.9	384.6 ± 32.2 [a]	397.0 ± 42.3	319.8 ± 38.5 [a b]	245.9 ± 50.2 [a b c]
**Serum phosphorus (mg/dL)**	6.0 ± 1.1	5.0 ± 0.7	8.5 ± 2.7	11.0 ± 10.3	11.6 ± 7.1	19.0 ± 6.2 [a]
**Serum glucose (mg/dL)**	268.8 ± 64.9	215.3 ± 41.5	180.3 ± 53.6	199.3 ± 88.2	177.4 ± 88.2	114.5 ± 70.9
**Serum BUN (mg/dL)**	18.4 ± 1.5	33.3 ± 6.	162.1 ± 47.6 [a b]	158.6 ± 33.8 [a]	207.5 ± 84.0 [a b]	270.7 ± 95.8 [a b c]
**Serum creatinine (mg/dL)**	0.60 ± 0.04	0.92 ± 0.15	3.26 ± 0.91 [a b]	3.81 ± 0.98 [a b]	4.00 ± 1.62 [a b]	4.31 ± 1.55 [a b]
**Serum uric acid (mg/dL)**	0.74 ± 0.17	0.65 ± 0.06	0.90 ± 0.24	1.53 ± 1.53	2.34 ± 0.56 [a b]	3.74 ± 1.49 [a b c]
**Serum sodium (mmol/L)**	143 ± 1.0	143.5 ± 2.6	141.3 ± 3.1	153.7 ± 13.4	138.6 ± 6.7	138.2 ± 13.3
**Serum potassium (mmol/L)**	4.8 ± 0.3	4.9 ± 0.3	5.7 ± 0.3	11.2 ± 7.3 [a b]	9.7 ± 0.9 [a b c]	15.8 ± 9.8 [a b]
**Serum chloride (mmol/L)**	100.2 ± 2.2	100.5 ± 3.4	96.6 ± 4.3	105.7 ± 15.0	92.2 ± 3.1 [a b d]	89.0 ± 7.7 [a b c d]

Data expressed as mean ± standard deviation. BUN, blood urea nitrogen.

[a] $p<0.05$ vs. control group; [b] vs. group 1; [c] vs. group 2; [d] vs. group 3A.
